# High-throughput and simultaneous quantitative analysis of homocysteine–methionine cycle metabolites and co-factors in blood plasma and cerebrospinal fluid by isotope dilution LC–MS/MS

**DOI:** 10.1007/s00216-016-0003-1

**Published:** 2016-10-18

**Authors:** Seu Ping Guiraud, Ivan Montoliu, Laeticia Da Silva, Loïc Dayon, Antonio Núñez Galindo, John Corthésy, Martin Kussmann, Francois-Pierre Martin

**Affiliations:** Nestlé Institute of Health Sciences SA, Campus EPFL, Innovation Park, CH-1015 Lausanne, Switzerland

**Keywords:** Methionine pathway, One-carbon metabolism, LC–MS/MS, High throughput, Plasma, Cerebrospinal fluid

## Abstract

The methionine cycle is a key pathway contributing to the regulation of human health, with well-established involvement in cardiovascular diseases and cognitive function. Changes in one-carbon cycle metabolites have also been associated with mild cognitive decline, vascular dementia, and Alzheimer’s disease. Today, there is no single analytical method to monitor both metabolites and co-factors of the methionine cycle. To address this limitation, we here report for the first time a new method for the simultaneous quantitation of 17 metabolites in the methionine cycle, which are homocysteic acid, taurine, serine, cysteine, glycine, homocysteine, riboflavin, methionine, pyridoxine, cystathionine, pyridoxamine, *S*-adenosylhomocysteine, *S*-adenosylmethionine, betaine, choline, dimethylglycine, and 5-methyltetrahydrofolic acid. This multianalyte method, developed using ultra-performance liquid chromatography–tandem mass spectrometry (UPLC–MS/MS), provides a highly accurate and precise quantitation of these 17 metabolites for both plasma and cerebrospinal fluid metabolite monitoring. The method requires a simple sample preparation, which, combined with a short chromatographic run time, ensures a high sample throughput. This analytical strategy will thus provide a novel metabolomics approach to be employed in large-scale observational and intervention studies. We expect such a robust method to be particularly relevant for broad and deep molecular phenotyping of individuals in relation to their nutritional requirements, health monitoring, and disease risk management.

**Electronic supplementary material:**

The online version of this article (doi:10.1007/s00216-016-0003-1) contains supplementary material, which is available to authorized users.

## Introduction

**Fig. 1:**
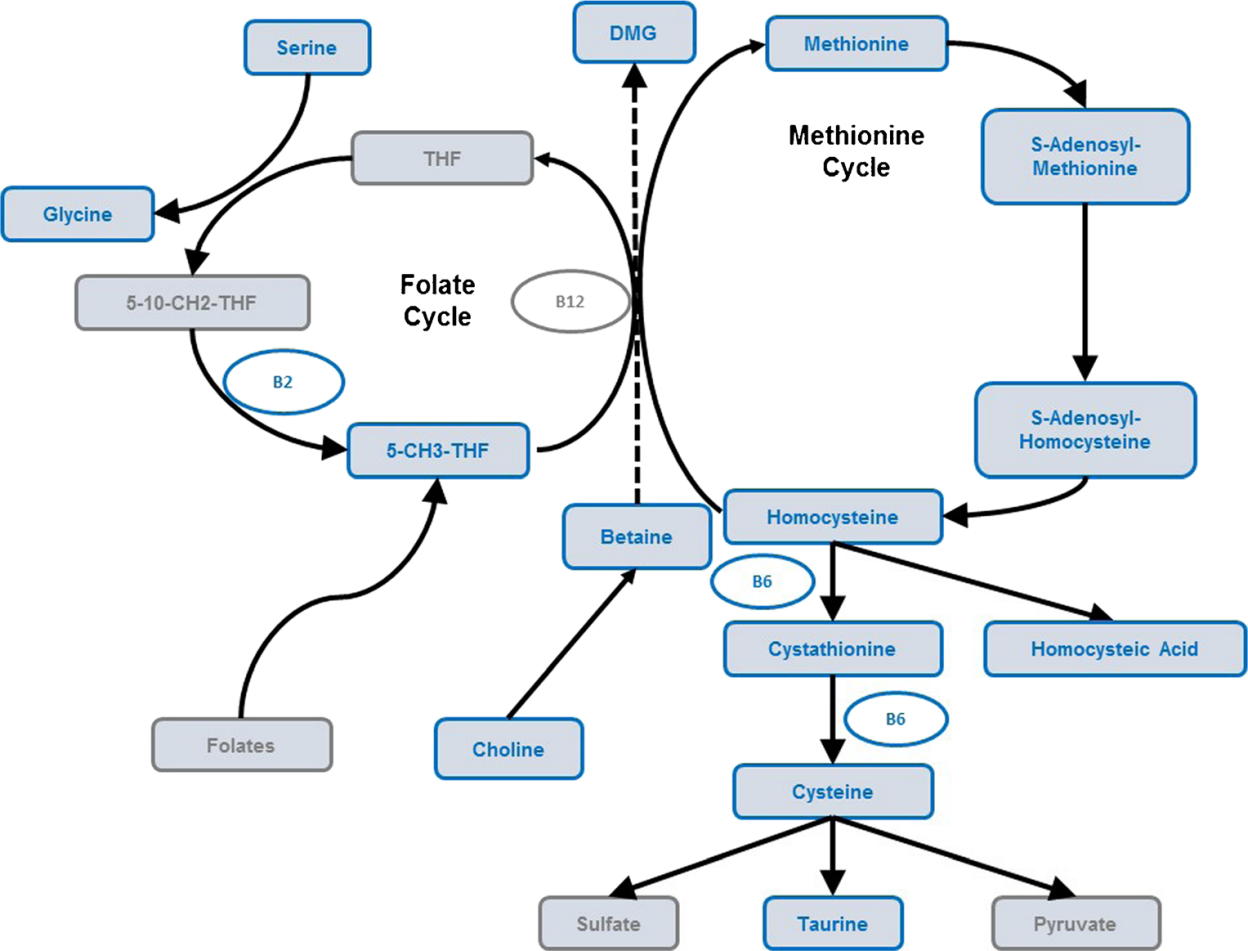
Metabolic pathway involving the measured (*blue*) metabolites from the homocysteine–methionine cycle

The coupling of the increased percentage of elderly in the world’s population and the incidences of chronic diseases is stimulating renewed interest in understanding the role of genetics, environmental factors, and their interactions with individual susceptibility to disease [1]. However, the determination of nutritional requirements for optimizing metabolism for an individual or population remains challenging due to the complexity of food macro- and micronutrient composition, intersubject variability in physiological responses, environmental, and genetic factors [2]. Over the last two decades, the era of omics technologies has provided an innovative paradigm for exploring physiological and pathological processes through broad and deep biological phenotyping [3]. The methionine cycle is among the many central pathways that contribute to human health. Dysfunction of this pathway has been linked to cardiovascular disease, mild cognitive decline, vascular dementia, and Alzheimer’s disease [4]. In addition, co-factors derived from diet are crucial for proper functioning of the methionine cycle. A key product of this pathway, *S*-adenosylmethionine (SAM), is itself a co-factor and substrate for methylation of DNA, protein, and RNA as well as biosynthetic reactions of key brain metabolites [5]. Hence, optimizing this pathway through nutrition may protect the brain from damage and reduce the risk of cardiovascular events. Monitoring the activity of this pathway in response to nutrition through metabolomics analysis (i.e., nutritional metabolomics) would generate a more comprehensive understanding of the interplay between host, environment, and nutrient interactions [6]. In particular, mass spectrometry (MS)-based metabolomics methods have demonstrated robust, accurate, and precise quantitation of several homocysteine–methionine cycle biomarkers in diverse biological matrices [7–10]. However, no single and high-throughput method currently exists to monitor both metabolites and co-factors in the methionine pathway. Most available analytical methods for methionine pathway metabolites have so far deployed multiple and typically separate chromatographic, derivatization, and/or detection schemes to identify and quantify methionine, sulfur-containing amino acids, homocysteine (HCy) and cysteine, *S*-adenosylmethionine/*S*-adenosylhomocysteine (SAM/SAH), and B vitamins [11–15]. To address this limitation, we have recently developed a new method for the simultaneous quantitation of 13 metabolites and co-factors from the so-called methionine pathway by liquid chromatography–tandem mass spectrometry (LC–MS/MS) in red blood cells [16]. In the present contribution, we introduce a further development of the method to enable a highly accurate and precise quantitation of 17 metabolites in plasma, including homocysteic acid (HA), taurine, serine, cysteine, glycine, homocysteine, riboflavin, methionine, pyridoxine, cystathionine, pyridoxamine, SAH, SAM, betaine, choline, dimethylglycine (DMG), and 5-methyltetrahydrofolic acid (5-MTHF) (Fig. 1). This improved method is also based on a simple sample preparation, which combined to a short chromatographic run time ensures a high sample throughput. This novel analytical strategy was also successfully applied for cerebrospinal fluid (CSF) analysis. Thus, this method provides a novel metabolomics approach for large-scale observational and human intervention studies. We demonstrate its applicability by analyzing plasma and CSF samples from healthy elderly subjects and patients diagnosed with Alzheimer’s disease (AD).

## Materials and methods

### Materials

LC–MS-grade acetonitrile (ACN), LC-grade methanol (MeOH), formic acid, perfluoroheptanoic acid (PFHA), ascorbic acid, sodium hydroxide (NaOH) 0.1 M, hydrochloric acid (HCl), tris(2-carboxyethyl)phosphine (TCEP), dithiothreitol (DTT), and ammonium acetate (NH_4_OAc) were purchased from Sigma-Aldrich Chemie GmbH (Buchs, Switzerland). Deionized water (*R* > 18 ΩM/cm, TOC < 10 ppb) was used throughout the experiments and produced by a Millipore-Q water system (Millipore, Bedford, MA, USA).

HA, taurine, serine, cystine, glycine, homocystine (HCy2), riboflavin, methionine, pyridoxine, cystathionine, SAH, pyridoxamine, SAM, DMG, choline, betaine, 5-MTHF, taurine-^13^C_2_, glycine-d_2_, riboflavin-dioxopyrimidine-^13^C_4_^15^N_2_, and methionine-d_3_ standards were purchased from Sigma-Aldrich Chemie GmbH.

HA-d_4_, serine-d_3_, pyridoxine-d_2_, cystathionine-d_4_, SAM-d_4_, DMG-d_6_, choline-d_9_, and betaine-d_11_ were purchased from CDN Isotopes (Pointe-Claire, Quebec, Canada). Cystine-d_4_, homocystine-d_8_, and pyridoxamine-d_3_ were purchased from Cambridge Isotopes Laboratories (Andover, MA, USA). SAH-d_4_ was purchased from Cayman Chemical (Ann Arbor, MI, USA). 5-MTHF-^13^C_5_ was purchased from Merck (Schaffhausen, Switzerland).

### Preparation of the individual standard and internal standard solutions

Standard solutions were prepared individually in 0.1 M HCl (or in 10 mmol/L NH_4_OAc, 0.1 M NaOH, or in a mixture of MeOH/H_2_O depending on their solubility). Only 5-MTHF was dissolved in a mixture of 10 mmol/L NH4OAc with 10 % ascorbic acid and 2 % DTT in H_2_O to prevent oxidation. The same dilution solvents were used for individual internal standard (IS) stock solutions (Table S1, see Electronic Supplementary Material (ESM)). Standard and IS solutions were stored at −20 °C for up to 3 months.

### Calibration curves and QC samples

Calibration standards were prepared by dilution of standard stock solutions in ACN/H_2_O (5/95; *v*/*v*) to obtain seven calibration solutions (calibrants 1 to 7) in micromolar range for HA, taurine, serine, cystine, glycine, methionine, HCy2, DMG, betaine, and choline and in nanomolar range for riboflavin, pyridoxine, cystathionine, pyridoxamine, SAH, SAM, and 5-MTHF as described in Table S2 (see ESM). An IS working solution was prepared by dilution of IS standard stock solutions in ACN/H_2_O (5/95; *v*/*v*) (see ESM Table S3) and stored at −20 °C for up to 3 months. Calibration samples were prepared with each batch of samples and injected at both the beginning and the end of the sample sequence. Fifty microliters of each calibration solution was pipetted and transferred into a 1.5-mL microcentrifuge tube. A volume of 10 microliters of IS working solution was then added to these solutions. Fifty microliters of TCEP (100 mg/mL) and then 140 μL of ACN/H_2_O (5/95; *v*/*v*) solution were furtherly added to the mixture. The tubes were placed on an autosampler rack and vortexed on a plate vortexer for 15 min at 1350 rpm. The solutions were transferred into vials and ready for LC–MS/MS analysis. Quality controls (QCs) at two different concentrations were added to each batch of samples. The low QC (at the same concentration as calibration level 3) and high QC (calibration level 6) samples were prepared using the same protocol as the calibration samples.

### LC–MS/MS instrumentation

The instrumental methodology was previously reported for red blood cell analysis [16] and is fully described in the ESM (Table S4 and Table S5). Briefly, a high-throughput method was developed using LC–MS/MS. Separation and analysis were performed on an Accela UHPLC 1250 Pump (Thermo Fisher Scientific Inc., Waltham, MA, USA) coupled to a TSQ Quantum Vantage triple quadrupole (Thermo Fisher Scientific Inc., Waltham, MA, USA) equipped with a heated electrospray ionization (H-ESI) source. Chromatographic separation was obtained using gradient elution on a reversed-phase UPLC XSelect HSST3 2.5 μm, 100 × 2.1 mm I.D. column (Waters Corporation, Milford, MA, USA). The injection volume was 10 μL and the total run time of analysis was 13 min.

### Human sample collection for method validation

The human sub-cohort was supplied from PrecisionMed, Inc. (CA, USA, protocol 8009). This sub-cohort comprises 12 individuals: 6 control subjects and 6 patients diagnosed with Alzheimer’s disease (AD). All the subjects are aged 50 years and older.

### Plasma and CSF sample collection and processing

Plasma or CSF frozen samples were thawed and vortexed for 10 s. A 50-μL volume of plasma or CSF samples was transferred by pipetting into a 1.5-mL microcentrifuge tube. Ten microliters of IS solution was added to plasma or CSF samples. Fifty microliters of TCEP solution and then 140 μL of methanol + 1 % FA were then added to the mixture. The tubes were placed in a multitube vortexer for 15 min at 1350 rpm at 4 °C and centrifuged at 14,500 rpm for 5 min. The supernatants were pipetted and filtered through a 0.22-μm filter and placed into vials for LC–MS/MS analysis.

### Matrix effects

Matrix effects were assessed by considering the post-extraction IS analyte spiking. Two sets of samples (five replicates for each set) were used: one containing the IS analyte added to an extracted matrix (post-extraction sample) and the other containing the IS analyte in the mobile phase solvent. Both sets of samples were spiked with the same concentration of IS analyte. Matrix effect values in percent were calculated using the following equation: matrix effect (%) = *B*/*A* * 100 (*A* = mean of external solution peak area, *B* = post-extraction sample peak area).

### Validation procedure

The validation procedure involves the following criteria: specificity and selectivity, limit of detection (LOD) and limit of quantitation (LOQ), linearity, trueness, precision (repeatability and intermediate precision), and recovery. Specificity and selectivity were assessed using the retention time and mass spectrum profile of pure standards and compared to the ones in unspiked CSF and plasma samples.

LOD and LOQ were evaluated based on the signal-to-noise ratio (*S*/*N*), at least above 3 for the LOD and 10 for the LOQ by injecting spiked plasma or CSF samples with the IS mixture of all analytes.

The calibration curves were constructed, at each concentration level, by calculating the chromatographic peak area ratio of the analyte and its IS for each metabolite.

Trueness and precision (repeatability and intermediate precision) were assessed by spiking plasma and CSF samples at low, medium, and high levels in six replicates for each level. For repeatability, six replicates were performed by the same operator on three separate occasions in a short period of time (less than 1 month). For intermediate precision, the same protocol was followed with additional analyses being carried out by two other individuals on a total of six separated occasions.

### Statistical analysis

Two-sample Kolmogorov–Smirnov (KS) tests were used to determine the statistical significance between gender distributions of metabolites. Uncorrected *P* values less than 0.05 were considered significant. The statistical software R v.3.2.2 [17] was used as a general platform for such analyses, and the package ggplot2 [18] was used for visualization.

## Results and discussion

The analytical method analyzes 17 metabolites involved in the homocysteine–methionine cycle. The previously described instrumental methodology used for the RBC matrix [16] was applied to the analysis of plasma and CSF samples. The analytical method extends the metabolite coverage, which now also includes betaine, DMG, choline, and 5-MTHF acid. Such an approach enables a more comprehensive assessment of homocysteine contribution via methionine synthase but also through betaine homocysteine methyltransferase (BHMT) to the methionine metabolism. The BHMT pathway is particularly active in both liver and kidney, which are the main organs for storage of large amounts of betaine [19]. This metabolite is actually distributed widely in plants and animals, particularly in seafood, and an inadequate dietary intake leads to hypomethylation of proteins, RNA, and DNA. Inadequate levels of betaine lead to disturbed hepatic protein metabolism, expressed by elevated homocysteine concentrations and decreased SAM concentrations in plasma [20]. Less well known are the correlations between betaine availability in plasma and its concentration in the CSF. Changes in the methionine cycle may be monitored by betaine–DMG conversion and the variations in 5-MTHF concentrations in plasma. In addition to betaine, choline is another important dietary methyl group donor particularly during folate deficiency [21]. Choline may enter the homocysteine–methionine cycle through its oxidation to betaine which is used as a substrate in the BHMT reaction that links choline and betaine to the folate-dependent methionine pathway. Our analytical method combines LC with MS/MS which provides a high specificity and sensitivity, as well as a fast run time (13 min). Compared to our previous methodology for RBC analysis, the current workflow for plasma and CSF does not require the same sample preparation, since there is no metabolite extraction from cells. Sample treatment was optimized for plasma samples and then applied to CSF samples. A simple and quick sample preparation including protein precipitation and chemical reduction was required for total cysteine and total homocysteine quantitation.

### Extraction method development

Homocysteine is found primarily in plasma in the form of free homocysteine (HCy), dimers, and analogue molecular forms bound by disulfide bridges to proteins or other thiol-containing compounds. The presence of these multiple forms complicates the development of reliable analytical methods. One common approach consists in the chemical reduction of all disulfide bonds to provide a total homocysteine measurement. Total homocysteine is therefore the sum of all homocysteine obtained from the reduction of these disulfide bonds; this also applies to total cysteine. Two reducing agents are frequently used for the reduction of disulfide bounds: DTT [22–26] and TCEP [27, 28]. We assessed both reducing agents and found that TCEP performed best in terms of stability and extraction efficiency. Indeed, TCEP provides a rapid chemical reduction of disulfide bonds at room temperature without any pH adjustments [29]. Yet, a concentration of 200 mM TCEP was found not sufficient to totally reduce the disulfide bonds, and the concentration was increased to 100 mg/mL [30]. Common solvents such as MeOH and ACN [31] are reported for protein precipitation for the analysis of total homocysteine in blood with or without the addition of acids such as FA. We found that MeOH + 1 % FA gave on overall the best mass spectral peak intensities and shapes.

### Chromatography and mass spectra

**Fig. 2:**
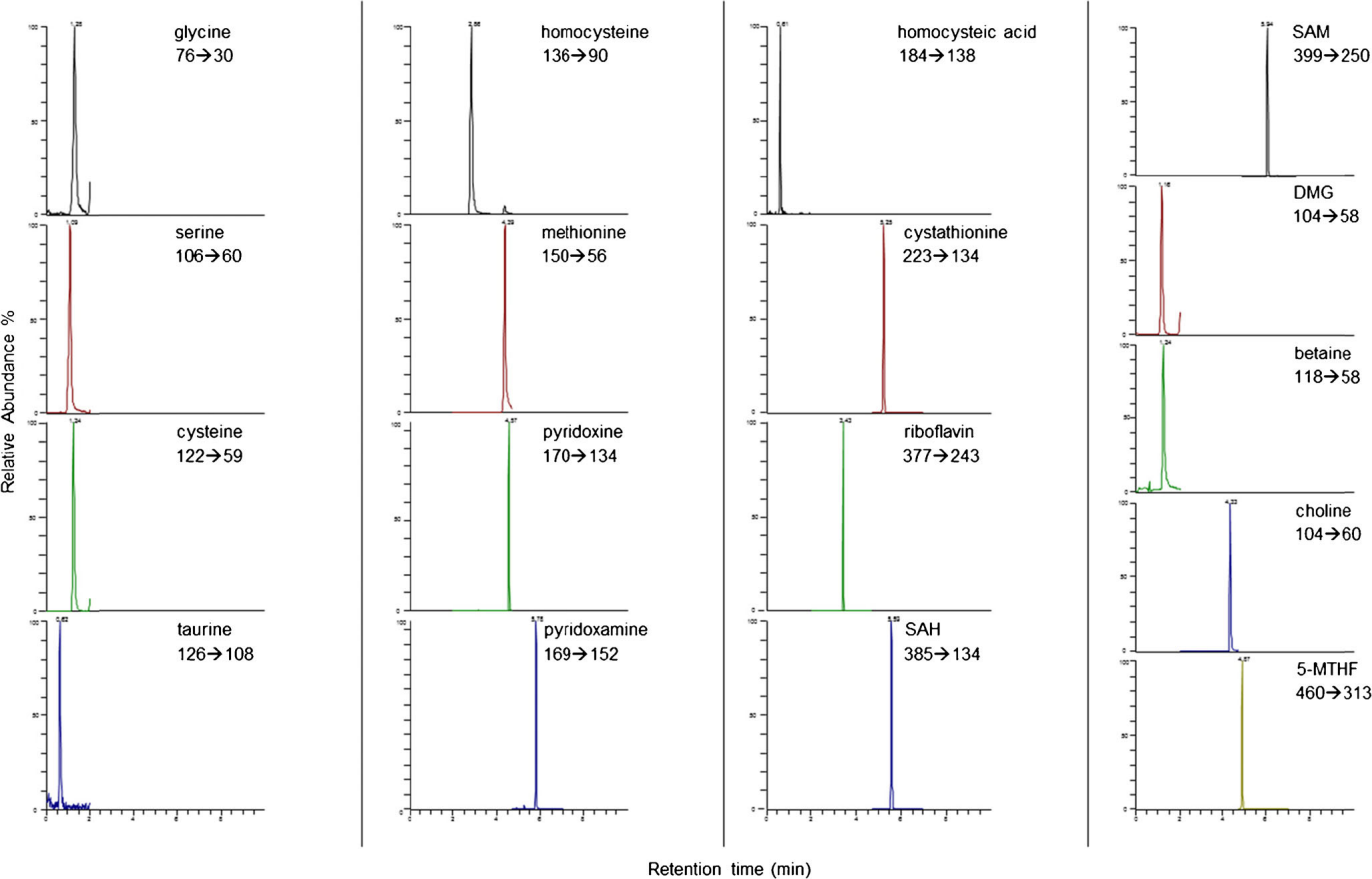
Typical SRM chromatograms obtained for spiked CSF samples at the medium level

The chromatographic method optimization is fully described in our previously published paper [16]. In this method, retention of the most polar compounds on a reversed-phase column was not possible without the use of ion-pairing agents. Typical chromatograms of a spiked CSF sample at the medium level are shown in Fig. 2. Optimal selected reaction monitoring (SRM) conditions were obtained in positive electrospray ionization mode and are reported in Table 1.

**Table 1:** Single reaction monitoring (SRM) transitions

Analyte	RT^a^ (min)	SRM transitions (*m*/*z*)	CE (eV)
Glycine	1.4	76 → 30	11
Glycine-d_2_	1.4	78 → 32	7
Serine	1.15	106 → 60	10
Serine-d_3_	1.15	109 → 63	11
Cysteine	1.4	122 → 59	20
Cysteine-d_2_	1.4	124 → 61	23
Taurine	0.6	126 → 108	11
Taurine-^13^C_2_	0.6	128 → 110	11
Homocysteine	2.85	136 → 90	10
Homocysteine-d_4_	2.85	140 → 94	10
Methionine	4.3	150 → 104	10
Methionine-d_3_	4.3	153 → 107	9
Pyridoxine	4.5	170 → 134	18
Pyridoxine-d_2_	4.5	172 → 136	19
Pyridoxamine	5.75	169 → 152	11
Pyridoxamine-d_3_	5.75	172 → 155	12
Homocysteic acid	0.6	184 → 138	10
Homocysteic acid-d_4_	0.6	188 → 142	9
Cystathionine	5.2	223 → 134	13
Cystathionine-d_4_	5.2	227 → 138	13
Riboflavin	3.4	377 → 243	15
Riboflavin-^13^C_4_^15^N_2_	3.4	383 → 249	13
SAH	5.6	385 → 134	21
SAH-d_4_	5.6	389 → 136	18
SAM	5.9	399 → 250	14
SAM-d_3_	5.9	402 → 250	13
DMG	1.25	104 → 58	12
DMG-d_6_	1.25	110 → 64	15
Betaine	1.4	118 → 58	25
Betaine-d_11_	1.4	129 → 66	31
Choline	4.35	104 → 60	17
Choline-d_9_	4.35	113 → 69	19
5-MTHF	4.9	460 → 313	15
5-MTHF-^13^C_5_	4.9	465 → 313	26

^a^RT retention time given as information only

### Validation procedures. Estimated LOD and LOQ

Due to endogenous levels of some analytes and incomplete reference material for all the analyzed metabolites, the LOD and LOQ were estimated by injecting plasma and CSF samples spiked by serial dilution of IS solution until reaching a *S*/*N* above 3 for the LOD and above 10 for the LOQ. Stable isotope-labeled compounds have the same physico-chemical properties and should therefore have the same analytical behavior as the analogue molecules (e.g., retention time, intensity). Estimated LOD and LOQ values from plasma and CSF samples spiked with IS solutions are reported in Table 2.

**Table 2:** Estimated limits of detection (LOD) and limits of quantitation (LOQ) values from plasma and CSF samples spiked with labeled internal standard (IS) solutions

Analyte	In plasma			In CSF		
	LOD	LOQ	Units	LOD	LOQ	Units
Homocysteic acid	0.5	1.0	μM	0.25	0.5	μM
Taurine	2.0	3.0	μM	4.0	5.0	μM
Serine	2.0	3.0	μM	0.5	1.0	μM
Total cysteine	0.1	0.5	μM	0.05	0.125	μM
Glycine	2.0	3.0	μM	1.0	2.0	μM
Total homocysteine	1.0	2.0	μM	0.125	0.25	μM
Riboflavin	1.0	2.0	nM	4.0	5.0	nM
Methionine	2.0	3.0	μM	0.5	1.0	μM
Pyridoxine	2.0	3.0	nM	1.5	2.0	nM
Cystathionine	0.5	1.0	nM	0.5	1.0	nM
SAH	0.1	0.5	nM	2.0	4.0	nM
Pyridoxamine	2.0	3.0	nM	0.25	0.5	nM
SAM	2.0	3.0	nM	2.0	4.0	nM
DMG	2.0	4.0	μM	0.1	0.4	μM
Betaine	0.05	0.125	μM	0.01	0.02	μM
Choline	0.02	0.05	μM	0.05	0.1	μM
5-MTHF	6.0	8.0	nM	2.0	4.0	nM

### Linearity of the calibration

We assessed whether to use solvent-based calibration curves or matrix-matched external calibration curves to accurately quantitate our 17 metabolites. Calibration curves were therefore performed in both solvent and sample matrix (plasma and CSF) using 7 calibration levels. The plasma or CSF calibration slopes differed by less than 16 % from the solvent-based calibration curves. Without IS correction, these differences would have been far higher in such complex matrices. These results show the importance of using isotopically labeled compounds as spiked IS to efficiently compensate for the matrix effect. For simplicity, only water-based calibration curves were used during this validation to accurately quantify these analytes in plasma or CSF samples.

The linearity of the calibration curves was assessed using seven calibration levels with five replicates each. The relationship between analyte response and concentration was linear in the solvent in the range mentioned in Table S2 (see ESM). For all compounds including the additional compounds, i.e., DMG, betaine, choline, and 5-MTHF, a linear regression model was used. A weighting factor of 1/*x* was applied for all compounds to achieve a linear model with the lowest relative errors at each level. All coefficients of determination (*r* ^2^) were found above 0.99. The acceptance criterion for each back-calculated standard concentration was ±15 % deviation from the nominal value except at the lowest level, i.e., level 1, which was set at ±20 %. All values were found within these ranges with a maximum of 2 excluded calibration points per curve.

### Trueness, precision, and recovery

Values for trueness and precision are summarized in Tables 3 and 4. For plasma samples, within-run precision (repeatability *r*) and between-run precision (intermediate reproducibility iR) CV values were found below 20 % for the lowest spiking level and below 15 % for the medium and high spiking levels, which are within the recommended limits [32]. For CSF samples, *r* and iR values were found below 16 % for the lowest spiking level and below 12 % for the medium and high spiking levels. Recoveries were found between 90 and 108 % for plasma samples and between 96 and 109 % for CSF samples, which are also within the recommended limits.

**Table 3:** Repeatability and intermediate trueness and precision values at three different concentration levels (*n* = 6 per day, per level) to determine accuracy of the developed method in plasma samples

Analyte	Targeted concentration	Mean ± SD	Units	Recovery ± RSD (%)	CV_*r*_ (%)	CV_iR_ (%)
Homocysteic acid	15.0	15.0 ± 0.8	μM	100.2 ± 2.1	6.2	7.7
	100.0	102.2 ± 3.9		102.2 ± 1.6	6.6	7.2
	200.0	199.6 ± 7.3		99.8 ± 1.5	6.3	6.8
Taurine	30.0	29.6 ± 2.8	μM	98.6 ± 3.8	12.5	14.7
	200.0	188.0 ± 14.3		94.0 ± 3.1	7.3	10.1
	400.0	370.0 ± 24.0		92.5 ± 4.8	5.7	8.3
Serine	30.0	30.1 ± 0.8	μM	100.3 ± 1.1	7.9	7.7
	200.0	203.4 ± 6.2		101.7 ± 1.2	3.2	4.3
	400.0	405.6 ± 11.6		101.4 ± 1.2	4.1	4.7
Total cysteine	15.0	16.1 ± 0.9	μM	107.6 ± 4.1	14.1	14.0
	100.0	101.5 ± 1.9		101.5 ± 0.8	7.9	7.5
	200.0	191.4 ± 12.5		95.7 ± 2.7	7.1	9.2
Glycine	30.0	28.6 ± 1.8	μM	95.3 ± 2.6	15.2	15.2
	200.0	197.9 ± 6.1		99.0 ± 1.3	5.1	5.6
	400.0	399.9 ± 15.8		100.0 ± 1.6	4.8	5.9
Total homocysteine	15.0	14.6 ± 0.4	μM	97.5 ± 1.0	2.9	3.7
	100.0	100.3 ± 1.7		100.3 ± 0.7	2.2	2.6
	200.0	197.0 ± 5.0		98.5 ± 1.0	6.2	6.2
Riboflavin	30.0	29.5 ± 3.1	nM	98.3 ± 4.3	18.0	19.5
	200.0	193.9 ± 10.2		97.0 ± 2.1	6.2	7.7
	400.0	399.5 ± 28.0		99.9 ± 2.9	8.6	10.5
Methionine	15.0	15.2 ± 0.4	μM	101.4 ± 1.1	6.1	6.2
	100.0	103.1 ± 2.4		103.1 ± 1.8	2.8	3.5
	200.0	202.7 ± 5.9		101.4 ± 1.2	3.6	4.4
Pyridoxine	15.0	14.4 ± 0.4	nM	96.0 ± 2.4	9.1	8.8
	100.0	100.8 ± 3.2		100.8 ± 1.3	4.0	4.8
	200.0	194.8 ± 9.3		97.4 ± 1.9	5.2	6.7
Cystathionine	30.0	30.8 ± 1.9	nM	102.5 ± 2.5	17.2	16.8
	200.0	194.7 ± 16.8		97.3 ± 3.5	9.7	12.4
	400.0	388.3 ± 26.5		97.1 ± 2.8	8.6	10.4
SAH	30.0	30.8 ± 3.1	nM	102.6 ± 4.1	17.9	19.1
	200.0	202.2 ± 11.2		101.1 ± 2.3	12.7	12.8
	400.0	398.8 ± 25.0		99.7 ± 2.6	11.7	12.4
Pyridoxamine	15.0	13.5 ± 1.0	nM	90.3 ± 6.2	16.0	16.4
	100.0	98.5 ± 4.4		98.5 ± 1.8	7.5	8.2
	200.0	195.1 ± 11.3		97.5 ± 2.4	9.5	10.4
SAM	30.0	26.5 ± 2.0	nM	88.4 ± 7.2	18.5	18.5
	200.0	200.6 ± 10.0		100.3 ± 2.0	12.1	12.1
	400.0	415.9 ± 36.7		104.0 ± 3.6	10.1	12.7
DMG	5.0	4.9 ± 0.1	μM	97.6 ± 1.4	5.2	5.1
	40.0	39.5 ± 2.0		98.7 ± 2.1	3.9	6.2
	80.0	79.5 ± 2.2		99.3 ± 1.1	2.4	3.6
Betaine	12.5	12.6 ± 0.9	μM	100.7 ± 2.9	7.0	9.5
	100.0	96.3 ± 2.3		96.3 ± 2.1	4.9	5.1
	200.0	196.3 ± 5.0		98.1 ± 1.0	3.0	3.7
Choline	5.0	4.9 ± 0.2	μM	98.8 ± 1.3	5.3	5.8
	40.0	39.4 ± 0.8		98.4 ± 0.8	4.2	4.3
	80.0	79.4 ± 1.7		99.2 ± 0.9	1.4	2.5
5-MTHF	50.0	47.8 ± 2.7	nM	95.7 ± 2.3	15.1	14.9
	400.0	393.0 ± 26.5		98.3 ± 2.7	12.8	13.5
	800.0	804.4 ± 26.8		100.5 ± 1.4	10.5	10.2

**Table 4:** Repeatability and intermediate trueness and precision values at three different concentration levels (*n* = 6 per day, per level) to determine accuracy of the developed method in CSF samples

Analyte	Targeted concentration	Mean ± SD	Units	Recovery ± RSD (%)	CV_*r*_ (%)	CV_iR_ (%)
Homocysteic acid	12.5	12.9 ± 0.3	μM	103.2 ± 1.8	6.7	6.5
	100.0	102.4 ± 4.2		102.4 ± 1.7	4.8	6.0
	200.0	200.7 ± 8.5		100.4 ± 1.7	3.9	5.5
Taurine	25.0	25.4 ± 0.5	μM	101.7 ± 0.9	8.8	8.3
	200.0	199.4 ± 10.6		99.7 ± 2.2	5.0	7.0
	400.0	386.5 ± 16.7		96.6 ± 1.8	3.1	5.2
Serine	25.0	24.5 ± 1.1	μM	98.1 ± 1.7	6.5	7.3
	200.0	202.6 ± 5.9		101.3 ± 1.2	3.8	4.6
	400.0	402.8 ± 14.3		100.7 ± 1.4	2.7	4.3
Total cysteine	12.5	12.7 ± 0.4	μM	101.3 ± 1.4	4.7	5.4
	100.0	102.8 ± 3.0		102.7 ± 1.2	3.2	4.2
	200.0	201.0 ± 4.9		100.5 ± 1.0	2.7	3.5
Glycine	25.0	25.2 ± 1.3	μM	100.7 ± 2.1	5.3	7.0
	200.0	201.7 ± 6.0		100.8 ± 1.2	3.4	4.3
	400.0	393.3 ± 8.3		98.3 ± 0.9	2.5	3.1
Total homocysteine	12.5	12.4 ± 0.6	μM	99.2 ± 1.9	2.6	5.3
	100.0	102.7 ± 1.8		102.7 ± 1.5	3.5	3.7
	200.0	202.4 ± 4.4		101.2 ± 0.9	2.2	2.9
Riboflavin	25.0	25.4 ± 2.8	nM	101.7 ± 4.5	16	18.3
	200.0	202.1 ± 7.8		101.0 ± 1.6	11.7	11.4
	400.0	402.5 ± 23.8		100.6 ± 2.4	7.8	9.2
Methionine	12.5	12.9 ± 0.6	μM	103.0 ± 1.8	4.9	6.3
	100.0	104.2 ± 4.2		104.2 ± 1.6	3.4	5.1
	200.0	203.9 ± 6.5		102.0 ± 1.3	3.4	4.5
Pyridoxine	12.5	12.6 ± 0.3	nM	100.9 ± 0.9	6.6	6.4
	100.0	102.3 ± 4.3		102.3 ± 1.7	3.9	5.5
	200.0	200.7 ± 6.8		100.4 ± 1.4	3.3	4.5
Cystathionine	25.0	24.1 ± 1.0	nM	96.5 ± 1.6	11.4	11.1
	200.0	213.9 ± 16.3		107.0 ± 3.1	7.2	10.1
	400.0	433.1 ± 28.4		108.3 ± 4.7	7.1	9.2
SAH	50.0	48.1 ± 3.5	nM	96.3 ± 3.0	12.8	13.8
	400.0	403.8 ± 14.3		100.9 ± 1.4	6.9	7.2
	800.0	773.0 ± 18.5		96.6 ± 2.0	6.9	6.7
Pyridoxamine	12.5	12.1 ± 0.4	nM	96.8 ± 2.0	9.9	9.5
	100.0	101.3 ± 3.5		101.3 ± 1.4	7.8	7.9
	200.0	200.0 ± 8.8		100.0 ± 1.8	7.6	8.2
SAM	50.0	51.1 ± 4.4	nM	102.1 ± 3.5	13.9	15.3
	400.0	406.2 ± 19.1		101.6 ± 1.9	8.5	9.1
	800.0	807.2 ± 41.6		100.9 ± 2.1	5.9	7.5
DMG	5.0	5.0 ± 0.2	μM	99.8 ± 1.2	3.1	4.1
	40.0	40.6 ± 1.1		101.5 ± 1.1	3.2	4.0
	80.0	80.8 ± 2.1		101.0 ± 1.0	2.8	3.6
Betaine	5.0	5.2 ± 0.2	μM	102.5 ± 1.5	5.2	6.1
	40.0	41.1 ± 1.0		102.8 ± 1.7	3.2	3.8
	80.0	80.5 ± 2.6		100.7 ± 1.3	2.3	3.8
Choline	5.0	5.0 ± 0.1	μM	100.2 ± 1.2	4.9	5.3
	40.0	40.7 ± 1.0		101.8 ± 1.0	3.1	3.8
	80.0	79.9 ± 1.0		99.9 ± 0.5	2.9	3.0
5-MTHF	50.0	49.5 ± 3.9	nM	99.1 ± 3.2	12.5	13.9
	400.0	408.0 ± 7.7		102.0 ± 0.8	5.7	5.5
	800.0	818.2 ± 29.8		102.3 ± 1.5	3.8	5.0

### Matrix effects

Matrix effects may occur in any LC–MS analysis because of the presence of co-eluting compounds in the sample matrix, especially in complex matrices such as plasma. The use of stable isotope-labeled related compounds as IS is a well-known efficient approach to overcome and correct matrix effects and therefore improve the method’s accuracy [33]. Although stable isotope-labeled compounds were used in our method, we still evaluated ion suppression and enhancement. Besides, ion suppression may still occur when using ion pairing such as heptafluorobutyric acid. Quantitative measurements for estimating the matrix effects are presented in Table S6 in the ESM.

### Application to biological samples

**Fig. 3:**
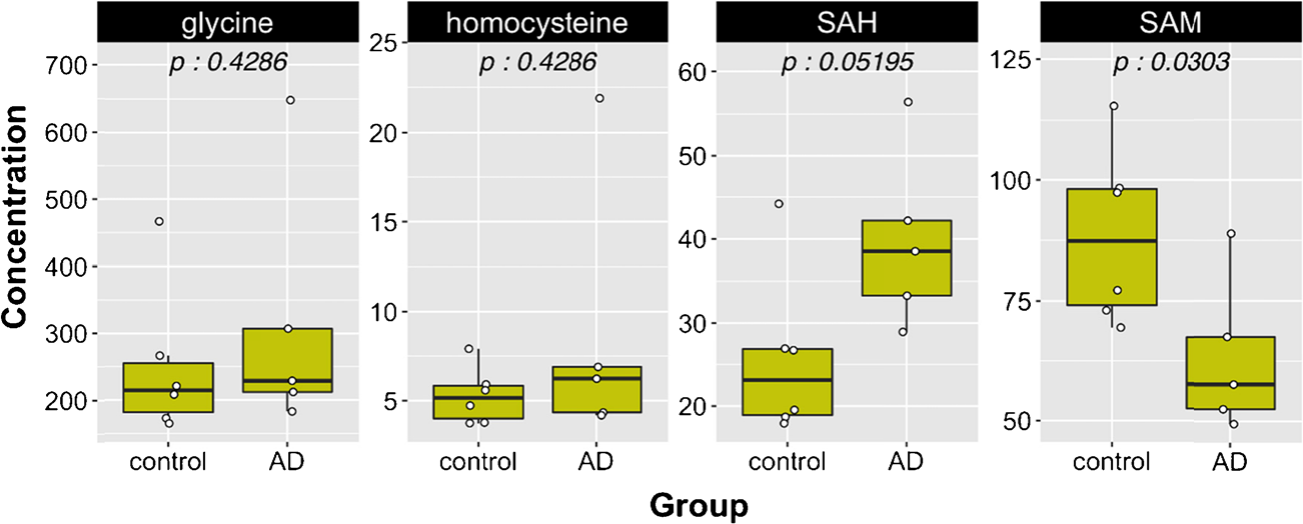
Plots in plasma samples. Concentrations for glycine and total homocysteine are in micromolars and for SAH and SAM in nanomolars. *P* values are reported

**Fig. 4:**
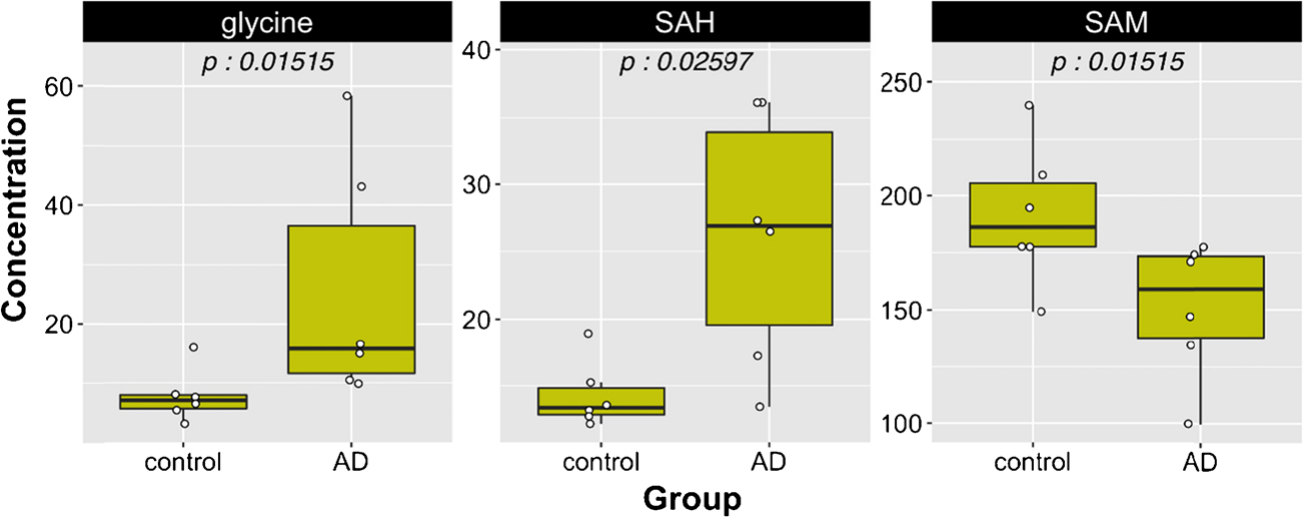
Plots in CSF samples. Concentrations for glycine are in micromolars and for SAH and SAM in nanomolars. *P* values are reported

The homocysteine–methionine cycle is of particular importance for cognitive function and Alzheimer’s disease. For instance, an imbalance of this metabolic pathway, marked by hyperhomocysteinemia and/or an altered SAM/SAH ratio, is a hallmark of memory loss and cognitive decline in elderly populations [34, 35] This loss of metabolic homeostasis may result from deficiency in metabolites and co-factors—such as vitamin B_12_ or folic acid—directly and indirectly involved in the methylation of homocysteine. An increased interest exists to decipher their role as potential biomarkers of neurodegeneration in Alzheimer’s disease [36–38]. The robustness of our method for the sensitive detection and quantitation of the 17 metabolites and co-factors of methionine metabolism was assessed in biological materials collected from healthy donors and patients diagnosed with Alzheimer’s disease. We reported in Table 5 plasma and CSF concentrations for the 17 metabolites in control and AD subjects. In blood plasma and CSF, the AD patients showed a significantly higher concentration of SAH compared to healthy subjects, but a lower circulating concentration of SAM (Figs. 3 and 4), a feature that was reported previously by other authors in several larger clinical studies using different and complementary assays [39–41]. However, despite the lack of statistical significance, our analysis also showed a trend toward a higher plasma level of homocysteine in those affected patients. In addition, a significant increase in glycine concentration in CSF was observed in AD patients, an observation that was confirmed by a similar trend in plasma samples. This metabolic pattern has also been reported previously by other authors using a targeted analytical method for amino acids [42]. Therefore, this application to real biological samples, although limited due to the number of subjects, demonstrated the importance of capturing sensitive metabolic readouts and contributed to obtain complete individual clinical phenotypes in plasma and CSF. Knowledge of the metabolites and co-factor distribution between plasma and CSF may yield information to better understand the impact of metabolic dysregulation and vitamin deficiencies/insufficiencies in the context of aging, cardiometabolic, and cognitive health research.

**Table 5:** Plasma and CSF concentrations of 17 metabolites in control and AD subjects (mean ± SD)

Analyte	Plasma		CSF		Units
	Control (*n* = 6)	AD (*n* = 6)	Control (*n* = 6)	AD (*n* = 6)	
	Mean ± SD	Mean ± SD	Mean ± SD	Mean ± SD	
Homocysteic acid	<LOD	<LOD	<LOD	<LOD	μM
Taurine	81.5 ± 30.9	71.6 ± 18	27.8 ± 4.7	24.0 ± 4.8	μM
Serine	79.6 ± 15.7	85.8 ± 19.4	25.4 ± 4.8	32.9 ± 14.5	μM
Total cysteine	144.4 ± 9.9	153.2 ± 32.9	1.1 ± 0.4	2.4 ± 2.2	μM
Glycine	250.0 ± 112.1	339.3 ± 180.6	7.8 ± 4.4	25.6 ± 20.3	μM
Total homocysteine	5.3 ± 1.6	8.1 ± 6.9	<LOQ	<LOQ	μM
Riboflavin	68.2 ± 66.6	21.9 ± 30.7	6.7 ± 5.3	12.9 ± 6.5	nM
Methionine	16.9 ± 2.8	16.8 ± 3.4	3.7 ± 0.4	5.3 ± 1.6	μM
Pyridoxine	<LOD	<LOD	<LOD	<LOD	nM
Cystathionine	116.7 ± 34.2	175.7 ± 112.3	46.0 ± 36.7	42.0 ± 32.9	nM
SAH	25.7 ± 9.9	40.9 ± 9.8	14.3 ± 2.5	26.1 ± 9.4	nM
Pyridoxamine	<LOD	<LOD	<LOD	<LOD	nM
SAM	88.5 ± 18.1	68.5 ± 19.4	191.4 ± 31.1	150.7 ± 30.2	nM
DMG	<LOQ	<LOQ	<LOQ	<LOQ	μM
Betaine	45.9 ± 14.5	45.8 ± 17.0	<LOD	<LOD	μM
Choline	11.2 ± 3.5	11.6 ± 3.7	3.0 ± 0.7	3.5 ± 0.5	μM
5-MTHF	56.6 ± 23.3	191.9 ± 363.5	60.9 ± 11.8	57.4 ± 18.8	nM

## Conclusions

A precise evaluation of changes in the homocysteine–methionine pathway is key to understand the relation among diet, nutrition and metabolic requirements, and cardiometabolic or neurological disorders. Quantitation of elements of this pathway is often covered by several analytical methods that just cover it partially. To fill this analytical gap, we have developed a robust, high-throughput LC–MS/MS method for the quantitation of 17 key metabolites embedded into the homocysteine–methionine metabolism. The approach described here provides a novel analytical tool allowing scientists to capture in one single analysis changes in the homocysteine–methionine pathway and their co-factors in plasma and CSF samples. The proposed analytical method does this with a high level of accuracy and reproducibility, making it especially interesting for large epidemiologic studies.

## Electronic supplementary material

Below is the link to the electronic supplementary material.

ESM 1 (PDF 278 kb)
