# Supplementary material for: High-throughput and simultaneous quantitative analysis of homocysteine–methionine cycle metabolites and co-factors in blood plasma and cerebrospinal fluid by isotope dilution LC–MS/MS
Source: Anal Bioanal Chem. 2016 Oct 18;409(1):295–305. doi: 10.1007/s00216-016-0003-1 (PMC5203846; doi:10.1007/s00216-016-0003-1)
Supplement: Supplementary file 1 — (PDF 278 kb) [file 216_2016_3_MOESM1_ESM.pdf]

Analytical and Bioanalytical Chemistry

Electronic Supplementary Material

High-throughput and simultaneous quantitative analysis of homocysteine–methionine cycle metabolites and co-factors in blood plasma and cerebrospinal fluid by isotope dilution LC–MS/MS

Seu Ping Guiraud, Ivan Montoliu, Laeticia Da Silva, Loïc Dayon, Antonio Núñez Galindo, John Corthésy, Martin Kussmann, Francois-Pierre Martin

Table S1 List of analyzed compounds and internal standard (IS) stock solutions using same dilution solutions. These solutions were stored at -20°C until use for 3 months

Analyte	Targeted concentration(μg/mL)	Concentration and solution type
homocysteic acid	2500.0	0.1 M HCl
taurine	2500.0	MeOH/H2O (10/90)
serine	2500.0	0.1 M HCl
cystine	2500.0	1 M HCl
glycine	2500.0	0.1 M HCl
homocystine	2500.0	0.1 M NaOH
riboflavin	250.0	0.1 M NaOH
methionine	2500.0	0.1 M HCl
pyridoxine	250.0	0.1 M HCl
cystathionine	250.0	0.1 M HCl
SAH	250.0	0.1 M HCl
pyridoxamine	250.0	0.1 M HCl
SAM	250.0	0.1 M HCl
DMG	2500.0	MeOH/H2O 10/90
betaine	2500.0	MeOH/H2O 10/90
choline	2500.0	MeOH/H2O 50/50
5-MTHF	250.0	NH_4OAc 10 mmol/L, ascorbic acid 10 %, DTT 2% in H_2O

Table S2 Working range of all metabolites for plasma and CSF analyses

Analyte	Conc. Range			Units
Homocysteic acid	2	-	250	µM
taurine	4	-	500	µM
serine	4	-	500	µM
cystine	2	-	250	µM
glycine	4	-	500	µM
homocystine	2	-	250	µM
riboflavin	4	-	500	nM
methionine	2	-	250	µM
pyridoxine	2	-	250	nM
cystathionine	4	-	500	nM
SAH	8	-	1000	nM
pyridoxamine	2	-	250	nM
SAM	8	-	1000	nM
DMG	0.8	-	100	µM
betaine	2	-	250	µM
choline	0.8	-	100	µM
5-MTHF	8	-	1000	nM

Table S3 Internal standard (IS) working solution concentrations for spiking

Analyte	Concentration	Units
homocysteic acid_d_4	1000	µM
taurine_$^{13}C_2$	2000	µM
serine-d_3	2000	µM
cystine_d_4	1000	µM
glycine_d_2	2000	µM
homocystine_d_8	1000	µM
riboflavin_$^{13}C_4{}^{15}N_2$	2	µM
methionine_d_3	1000	µM
pyridoxine_d_2	1	µM
cystathionine_d_4	2	µM
SAH_d_4	4	µM
pyridoxamine_d_3	1	µM
SAM_d_3	4	µM
DMG_d_6	400	µM
betaine_d_{11}	1000	µM
choline_d_9	400	µM
5-MTHF_$^{13}C_5$	4	µM

Table S4 Summary of HPLC settings

Injection volume	10 µL		
LC column temperature	Room temperature		
Mobile Phase	A – 5 mM PFHA in water<br>B – Acetonitrile		
Pre clean needle wash 1	ACN/H2O (50/50; v/v)		
Post clean needle wash 2	ACN/H2O (90/10; v/v)		
Mobile phase flow	0.4 mL/min		
Gradient	Time (min)	% A	% B
	0.0	95	5
	0.5	95	5
	8	5	95
	10.0	5	95
	11.0	95	5
	13.0	95	5
Total run time (min)	13.0 min		

Table S5 Summary of MS settings

Source type	ESI+
Spray voltage	4 kV
Vaporizer temperature	200 °C
Sheath gas pressure	10 units
Ion sweep gas pressure	0 units
Auxiliary gas pressure	5 units
Capillary temperature	350 °C
S-lens RF	used tune S-lens values
CID gas	Argon, quality 5.0, 1.5 mTorr
Scan width	0.5 s
Scan time	0.02 s

Table S6 Matrix effects % (ME) estimated in spiked plasma and CSF samples with labeled internal standards

**Analyte**	**ME % in plasma**	**ME % in CSF**
homocysteic acid	120.2	32.1
taurine	105.9	10.6
serine	-11.4	-24.5
total cysteine	2.4	-22.7
glycine	-10.1	-19.7
total homocysteine	6.5	-7.0
riboflavin	25.6	-3.0
methionine	-2.8	7.3
pyridoxine	-11.9	-8.6
cystathionine	-0.5	-9.1
SAH	-6.4	-3.8
pyridoxamine	0.7	-19.6
SAM	-3.0	-6.3
DMG	-14.4	-30.0
betaine	-15.1	-26.3
choline	-35.7	-23.2
5-MTHF	-73.3	-36.9
